# Gratification vs. compensation: longitudinal evidence for the shifting motivations behind adolescent gaming disorder

**DOI:** 10.3389/fpsyt.2026.1938938

**Published:** 2026-10-23

**Authors:** Sylwia Starzec, Aleksandra Zajas, Natalia Michałkiewicz, Patrycja Kiszka, Lidia Kowalska, Paweł Strojny

**Affiliations:** 1 Doctoral School in the Social Sciences, Jagiellonian University, Kraków, Poland; 2 Institute of Applied Psychology, Faculty of Management and Social Communication, Jagiellonian University, Kraków, Poland

**Keywords:** adolescents, compensation, gaming disorder, gaming motivation, gratification, I-PACE, longitudinal study

## Abstract

**Introduction:**

Drawing on the Interaction of Person-Affect-Cognition-Execution (I-PACE) model, this longitudinal study examined the prospective associations between gaming motives and Gaming Disorder (GD) among adolescents. Specifically, we investigated whether gratification-oriented motives (Fantasy, Identity) predict subsequent GD symptoms and whether compensation-oriented motives (Coping, Escape, Introjected Regulation) emerge as consequences of GD.

**Methods:**

Data were collected from 120 secondary-school gamers at two time points separated by a three-month interval. Cross-lagged panel models were estimated separately for each gaming motive–GD pair to examine the directionality of associations over time.

**Results:**

The findings supported the gratification hypothesis, as higher baseline Fantasy and Identity motives predicted increased GD symptoms at follow-up. A bidirectional association emerged between Fantasy and GD, whereas higher initial GD symptoms predicted a subsequent decline in Identity motives. The compensation hypothesis received partial support: GD prospectively predicted increases in Coping and Introjected Regulation, while no significant cross-lagged associations were observed for Escape.

**Discussion:**

These findings suggest that gratification-oriented motives may contribute to the early development of GD, whereas increasing GD symptoms may reinforce specific compensation-oriented motives. The results highlight distinct, motive-specific dynamics in adolescent gaming and underscore the importance of distinguishing between gratification and compensation processes. From a practical perspective, assessing Fantasy and Identity motives as potential risk indicators and addressing emerging Coping and Introjected Regulation motives may inform more targeted school-based prevention and intervention strategies.

## 1 Introduction

Gaming motivations are central to understanding why individuals engage in video games and why some develop problematic gaming patterns, often described as Gaming Disorder (GD) (1), as they reflect the underlying psychological needs and regulatory functions that drive both the initiation and maintenance of gaming behavior. According to the World Health Organization (1), gaming disorder is characterized by a persistent or recurrent pattern of gaming behavior, manifested by impaired control over gaming, increasing priority given to gaming over other life activities, and continuation or escalation of gaming despite the occurrence of negative consequences. Most existing studies examining the link between gaming motivations and GD have relied on cross-sectional designs (2, 3), whereas longitudinal investigations remain relatively rare (1, 4–7). Such investigations are essential to establish the temporal ordering of associations between gaming motives and GD, although temporal precedence alone cannot determine whether these relationships are causal.

A particularly relevant framework for this purpose is the Comprehensive Gaming Motivation Model (8). Within this model, the Immersion/Escapism dimension—comprising Coping, Escape, Fantasy, Identity, and Introjected Regulation—has shown the most consistent associations with GD (2). Although not explicitly proposed by Király et al., on the basis of item content these factors can be meaningfully grouped into two functional categories. Gratification-oriented motivations (Fantasy, Identity) reflect engagement in gaming for positive reinforcement, for example, immersion in the game world and the experience of gaming as personally meaningful or identity-related. Compensation-oriented motivations (Coping, Escape, Introjected Regulation), in contrast, reflect engagement aimed at alleviating negative affect or compensating for real-life difficulties, such as stress relief, avoidance, or the need to meet internalized expectations. This distinction resonates with the Person–Affect–Cognition–Execution (I-PACE) model of addictive behaviors, which distinguishes between gratification-driven and compensation-driven engagement in gaming (9–11). Recent work has also explicitly interpreted Coping-, Escapism-, and Fantasy-related motives within the I-PACE framework as reflecting anticipated compensatory effects of gaming (12).

Our previous three-wave longitudinal study of emerging adults (13), with measurements separated by three-month intervals, suggested that compensation-oriented motivations, particularly Coping and Escape, were the primary longitudinal predictors of GD risk, while gratification-oriented motives did not emerge as prospective predictors. The use of this interval was consistent with classical dynamic accounts of motivation, according to which motivation reflects the current configuration of personal and situational forces and the changing activation of goals and means (14, 15). However, such a pattern may not generalize straightforwardly to adolescence. Adolescence involves ongoing and heterogeneous changes in reward processing, cognitive control, and goal-directed learning. In some contexts, greater sensitivity to salient rewards and increased exploration may make gratification-oriented motives particularly relevant; however, these tendencies vary substantially across individuals and are not inherently maladaptive (16–18). While adult-derived models emphasize a transition from positive to negative reinforcement (19, 20), prospective associations between gratification-oriented motives and GD risk may be particularly relevant in secondary-school populations. Emerging evidence indicates complex, age- and context-dependent patterns of association with GD risk (7, 21).

Taken together, these considerations suggest that the longitudinal role of gaming motivations in GD may vary across developmental periods. In the present study, we bridge the gap between adult-derived models and adolescent development. Guided by the I-PACE framework, we expected gratification-oriented motives (Fantasy, Identity) at Time 1 to be positively associated with GD risk at Time 2. Conversely, we expected GD risk at Time 1 to be positively associated with compensation-oriented motives (Coping, Escape, Introjected Regulation) at Time 2. At the same time, findings from our previous longitudinal study among emerging adults indicated that this pattern may not generalize straightforwardly across age groups. Accordingly, the present study was approached not only as a test of theoretically derived directional hypotheses, but also as an opportunity to examine whether the expected I-PACE-consistent pattern would be observed in adolescents, or whether a different pattern of prospective associations would be observed. On this basis, the following hypotheses were formulated:

H1: Coping, Escape, Introjected Regulation, Fantasy, and Identity at T1 and T2 will each correlate with the risk of GD at corresponding time points.
H2: Time 1 gratification-oriented motivations (Fantasy, Identity) will be positively associated with GD risk at Time 2 after accounting for GD risk at Time 1.
H3: Time 1 GD risk will be positively associated with compensation-oriented motivations (Coping, Escape, Introjected Regulation) at Time 2 after accounting for their respective Time 1 levels.

## 2 Method

### 2.1 Participants and procedure

Participants in the study were recruited by announcements sent out through the school’s communication system. Participants were recruited from multiple grade levels (grades 1–3) within a single school. Specific classroom identifiers were not recorded. The study was voluntary and unpaid, and written informed consent was obtained from both underage participants and their parents prior to participation. Only students who reported playing video games for at least one hour per week were admitted to the procedure. Participants completed the online questionnaires on their personal mobile devices during class under the supervision of research assistant and/or teacher. The procedure included two attention check items, and only those who passed both checks were retained for further analysis. To enable longitudinal matching while preserving anonymity, participants generated an anonymous code based on randomly selected personal information. The study was conducted in two waves, separated by a three-month interval (T1, T2), starting in December 2024. The present study is part of a larger longitudinal research program. Cross-sectional findings based on T1 data from the same adolescent project were previously reported by Michałkiewicz et al. (22). That study examined all 26 GMI motives as exploratory predictors of GD symptoms and subsequently tested cross-sectional indirect associations involving gaming rage and post-gaming disengagement. The present study reuses the T1 IGDS9-SF and GMI measurements of the longitudinally matched participants but addresses a distinct research question concerning prospective associations between GD risk and five motives from the GMI dimension. The previous publication did not include the T2 data or any of the longitudinal analyses reported here.

A total of 120 participants provided data that could be reliably linked across both waves and were therefore retained for the longitudinal analyses. At T1, a total of 320 participants completed the survey. Of these, 13 participants were excluded because they did not meet the eligibility criterion of playing video games, as indicated by the screening question, 40 participants were excluded because they failed attention-check items, and 7 were excluded because they provided invalid or incorrectly generated anonymous identification codes. Thus, 260 participants provided valid T1 data eligible for longitudinal matching.

At T2, 248 participants completed the survey. Of these, 17 were excluded for failing the attention checks and 16 for providing invalid anonymous identification codes, leaving 215 valid T2 cases. Of these, 120 participants could be reliably matched with their T1 data and constituted the final longitudinal sample. The remaining 140 valid T1 cases and 95 valid T2 cases could not be reliably linked across waves. Because matching relied exclusively on self-generated anonymous codes, it was not possible to distinguish true attrition from unsuccessful matching due to participants entering a different code at T2. A detailed participant flow diagram is provided in **Supplementary Figure S1**.

To assess potential selection bias, participants retained in the longitudinal sample (*n* = 120) were compared with participants who provided valid T1 data but were not retained (*n* = 140). No significant differences were found in age, gender, baseline GD symptoms, fantasy, identity, internal regulation, or escapism motives (all *p* >.05). However, retained participants reported higher baseline coping-motive scores than non-retained participants, *t(258*) = 2.27, *p* = .024, *d* = .28, 95% CI [.04,.53].

Participants were aged between 14 and 18 years (*M* = 15.45, *SD* = 0.97), including 71 girls (59%), 42 boys (35%), one non-binary participant, and six participants who preferred not to disclose their gender. No participants reached the IGDS9-SF research-based cut-off of ≥ 36 for disordered gaming at either T1 or T2.

### 2.2 Measurements

#### 2.2.1 Risk of gaming disorder

The risk of GD symptoms was measured with the Internet Gaming Disorder Scale – Short Form; IGD9-SF (23) and will hereafter be referred to simply as GD for the sake of brevity. The scale consists of 9 items reflecting the DSM-5 criteria for Internet Gaming Disorder. Participants rated each item on a 5-point Likert scale ranging from 1 (“never”) to 5 (“very often”). For descriptive purposes, we also considered the research-based cut-off of ≥ 36 proposed by Pontes and Griffiths (23) for classifying disordered versus non-disordered gaming in research contexts. This cut-off was used as a screening indicator and not as a clinical diagnosis. Internal consistency in the current study was satisfactory, both at T1: *α* = .79, and at T2: *α* = .83.

#### 2.2.2 Gaming motivation

Gaming motivation was assessed using the Gaming Motivation Inventory; GMI (8). The inventory measures several motivational dimensions of gaming, including Coping, Escape, Introjected Regulation, Fantasy, and Identity. Items were rated on a 7-point Likert scale from 1 (“does not correspond at all”) to 7 (“corresponds exactly”). Internal consistency in the current sample was mostly satisfactory: Coping at T1: *α* = .77, Coping at T1: *α* = .82, Escape at T1: *α* = .91, Escape at T2: *α* = .94, Introjected Regulation at T1: *α* = .61, Introjected Regulation at T2: *α* = .75, Fantasy at T1: *α* = .83, Fantasy at T2: *α* = .84, Identity at T1: *α* = .88, Identity at T2: *α* = .87.

#### 2.2.3 Other measures

The following instruments were also administered but were not included in the present analyses: Gaming Involvement Scale (24), Gaming Impact Scale (25), Post-Gaming Return Questionnaire (26), Rage in Gaming Scale (27), School Climate (Dual School Climate and School Identification Measure–Student; SCASIM-St15 (28). Further measures included school performance (self-reported grades and perceived achievement across subjects) and a brief health screening for comorbidities (prior diagnoses of depression, anxiety disorder, or ADHD). Participants also provided demographic data (age, gender, country, hometown size, socioeconomic status, parental education) and rated their response honesty on a single item.

### 2.3 Statistical approach

All statistical analyses were conducted using R with the *lavaan* and *semTools* packages. There was no missing data across waves. To examine the longitudinal associations between gaming motivations and gaming disorder risk, we employed cross-lagged panel analysis with item-parceling methodology, following the methods described by Wang et al. (29). The item-parceling approach was chosen to analyze cross-lagged effects in a two-wave study, because a traditional cross-lagged panel model with scale mean scores would be saturated (*df* = 0) and therefore not produce model fit indices. Item parceling increases model reliability and fit by aggregating items into parcels used as manifest indicators for latent constructs. In this case, the nine IGD items were randomly allocated to three parcels containing three items each. The items of GMI motivations with four or five items, namely Fantasy, Escape, and Identity, were randomly allocated to two parcels. The items of Coping and Introjected Regulation, which consisted three items each, were allocated to one parcel. The same parcel structure was used at T1 and T2. Where model fit indices are reported, values represent the average across all 100 model replications. The p-values provided for parameter estimates are based on approximate calculations, reflecting the averaged nature of the parcel-based analysis. Longitudinal measurement-invariance tests were not conducted.

## 3 Results

### 3.1 Hypothesis 1: associations between coping, escape, introjected regulation, fantasy, identity, and gaming disorder risk at T1 and T2

Consistent with H1, GD risk was positively correlated with all five motivational factors at both waves (see **Table 1**). Overall, H1 was supported: associations between GD risk and the motivational factors were consistently moderate to large, with the strongest links observed for Introjected regulation (both waves) and Fantasy (especially at T2). Means and standard deviations for each variable are reported in **Table 1**.

**Table 1:** Means, standard deviations, and correlations among study variables at T1 (N = 120) and T2 (N = 120).

	Variable	*M*	*SD*	1	2	3	4	5	6
T1	1. GD risk	1.82	.55	—					
	2. Coping	3.97	1.50	.52	—				
	3. Escape	3.25	1.71	.51	.66	—			
	4. Fantasy	3.02	1.64	.46	.61	.64	—		
	5. Identity	2.59	1.40	.53	.54	.29	.54	—	
	6. Introjected regulation	1.64	.82	.60	.45	.33	.40	.60	—
T2	1. GD risk	1.79	.57	—					
	2. Coping	3.92	1.63	.52	—				
	3. Escape	3.31	1.77	.51	.77	—			
	4. Fantasy	3.11	1.67	.61	.70	.65	—		
	5. Identity	2.60	1.41	.53	.59	.33	.58	—	
	6. Introjected regulation	1.81	1.04	.60	.48	.40	.58	.60	—

All correlations were significant at *p* <.01.

### 3.2 Hypothesis 2: prospective associations of time 1 gratification-related motivations with gaming disorder risk at time 2

#### 3.2.1 Fantasy

The cross-lagged analysis including GD risk and Fantasy demonstrated acceptable fit, χ²(29) = 64.708, *p* = .012, CFI = .951, TLI = .923, RMSEA = .099 (90% CI [.063,.133]), and SRMR = .049. Fantasy at Time 1 was prospectively associated with higher GD risk at Time 2 (*b* = .445, *SE* = .229, *p* <.001). The autoregressive paths were significant for Fantasy (*b* = 2.057, *SE* = 1.038, *p* <.001) and significant for GD (*b* = 1.648, *SE* = .586, *p* <.001). Cross-lagged effect of Fantasy at Time 1 on GD at Time 2 was significant (*b = .*445, *SE* = .229, *p* <.001), but this was not the case for the effect of GD at Time 1 on Fantasy at Time 2 (*b* = .162, *SE* = .321, *p* = .063). Cross-sectional correlations between Fantasy and GD were moderate to strong at both Time 1 (*r* = .565, *p* <.001) and Time 2 (residual correlation *r* = .593, *p* <.001).

#### 3.2.2 Identity

The model including Identity demonstrated good fit, χ²(29) = 54.251, *p* = .028, CFI = .966, TLI = .948, RMSEA = .083 (90% CI [.045,.119]), and SRMR = .044. Identity at Time 1 was prospectively associated with higher GD risk at Time 2 (*b* = .182, *SE* = .207, *p* = .007). Autoregressive effects were strong for both Identity (*b* = 2.250, *SE* = .632, *p* <.001) and GD (*b* = 1.636, *SE* = .532, *p* <.001). Interestingly, GD at Time 1 was prospectively associated with lower Identity at Time 2 (*b* = -.362, *SE* = .311, *p* <.001). Cross-sectional correlations were again substantial at both Time 1 (*r* = .604, *p* <.001) and Time 2 (residual correlation *r* = .859, *p* <.001).

#### 3.2.3 Summary of H2

Thus, the analyses supported Hypothesis 2. Both Fantasy and Identity motivations at Time 1 were prospectively associated with higher levels of GD symptoms at Time 2, after accounting for GD risk at Time 1. The reverse path from GD to Fantasy was not statistically significant, whereas GD at Time 1 was prospectively associated with lower Identity at Time 2.

### 3.3 Hypothesis 3: prospective associations of time 1 gaming disorder risk with compensation-related motivations at time 2

#### 3.3.1 Coping

The fit indices for the model including GD and Coping were mixed fit, χ²(16) = 45.542, *p* = .014, CFI = .940, TLI = .897, RMSEA = .121 (90% CI [.077,.165]), and SRMR = .049. Although CFI and SRMR were comparatively favorable, the elevated RMSEA and TLI below.90 indicate that estimates from this model should be interpreted cautiously. The autoregressive effects for both GD risk (*b* = 1.735, *SE* = .610, *p* <.001) and Coping (*b* = .878, *SE* = .166, *p* <.001) were significant. Cross-lagged path from Coping at T1 to GD risk at T2 was nonsignificant (*b* = -0.023, *SE* = .236, *p* = .792). Cross-lagged path from GD risk at T1 to Coping at T2 was significant and positive (*b* = 0.145, *SE* = .149, *p* <.001), indicating that GD risk at T1 was prospectively associated with higher Coping at T2 after accounting for Coping at T1. The correlation between GD and Coping at T1 was strong (*r* = .629, *p* <.001), and the residual correlation at T2 was moderate and significant (*r* = .363, *p* <.001).

#### 3.3.2 Escape

The cross-lagged analysis including GD and Escape showed mixed to poor model fit, χ²(29) = 87.62, *p* <.001, CFI = .934, TLI = .897, RMSEA = .128 (90% CI [.097,.160]), and SRMR = .069. The autoregressive paths were significant for GD risk (*b* = 1.887, *SE* = .676, *p* <.001) and Escape (*b* = 1.438, SE = .289, *p* <.001). Cross-lagged effects were nonsignificant: GD risk at T1 did not predict Escape at T2 (*b* = -.043, *SE* = .199, *p* = .442), nor did Escape at T1 predict GD risk at T2 (*b* = -.236, *SE* = .283, *p* = .085). Cross-sectional correlations between GD and Escape were moderate to strong and statistically significant at both time points (*r* = .596 at T1, *r* = .792 at T2, *p* <.001).

#### 3.3.3 Introjected regulation

The cross-lagged analysis including GD and Introjected Regulation demonstrated adequate fit, χ²(16) = 35.186, *p* = .044, CFI = .961, TLI = .932, RMSEA = .096 (90% CI [.048,.142]), and SRMR = .040. The autoregressive effect was significant for both GD risk (*b* = 1.807, *SE* = .679, *p* <.001), and for Introjected Regulation (*b* = .621, *SE* = .172, *p* <.001). Cross-lagged path from GD risk at T1 to Introjected Regulation at T2 was significant (*b* = .084, *SE* = .283, *p* = .030), but nonsignificant from Introjected Regulation at T1 to GD risk at T2 (*b* = -.114, *SE* = .291, *p* = .301). The correlation between GD and Introjected Regulation at T1 was strong (*r* = .691, *p* <.001), and the residual correlation at T2 also significant and strong (*r* = .738, *p* <.001).

#### 3.3.4 Summary of H3

The analyses provided partial support for Hypothesis 3. As expected, the risk of GD at Time 1 was prospectively associated with higher levels of Coping and Introjected Regulation at Time 2 after accounting for their respective Time 1 levels, but was not significantly associated with Escape. None of the reverse paths from the compensation-oriented motives at Time 1 to GD severity at Time 2 reached statistical significance. Estimated path coefficients for all models are reported in **Table 2**.

**Table 2:** Estimated path coefficients across all cross-lagged item-parcel models.

Motivation	Path	*B*	*Beta/r*	*SE*	*LLCI*	*ULCI*	*Min*	*Max*	*p*
Fantasy	GD T1 → GD T2	1.648	.754	.586	.500	2.797	1.259	2.368	<.001
	Fantasy T1 → Fantasy T2	2.057	.861	1.038	.023	4.090	1.605	2.997	<.001
	**Fantasy T1 → GD risk T2**	**.445**	**.205**	**.229**	**.005**	**.894**	**.244**	**.574**	**<.001**
	GD risk T1 → Fantasy T2	.162	.071	.321	-.467	.791	-.008	.377	.063
	Correlation GD ↔ Fantasy (T1)		.565	.084	.401	.730	.515	.640	<.001
	Residual correlation GD risk ↔ Fantasy (T2)		.593	.291	.023	1.163	.492	.808	<.001
Identity	GD risk T1 → GD risk T2	1.636	.803	.532	.565	2.651	1.301	2.140	<.001
	Identity T1 → Identity T2	2.250	.982	.632	.991	3.470	1.894	2.807	<.001
	**Identity T1 → GD risk T2**	**.182**	**.126**	**.207**	**.115**	**.594**	**-.058**	**.316**	**.007**
	GD risk T1 → Identity T2	-.362	-.157	.311	-.968	-.251	-.672	-.204	<.001
	Correlation GD risk ↔ Identity (T1)		.604	.074	.458	.746	.550	.660	<.001
	Residual correlation GD ↔ Identity (T2)		.859	.263	.325	1.357	.679	1.07	<.001
Coping	GD risk T1 → GD risk T2	1.735	.868	.610	.539	2.931	1.242	2.252	<.001
	Coping T1 → Coping T2	.878	.628	.166	.552	1.204	.823	.906	<.001
	Coping T1 → GD risk T2	-.023	-.010	.236	-.485	.440	-.245	.140	.792
	**GD risk T1 → Coping T2**	**.145**	**.104**	**.149**	**.086**	**.440**	**.100**	**.232**	**<.001**
	Correlation GD ↔ Coping (T1)		.629	.070	.493	.766	.572	.675	<.001
	Residual correlation GD ↔ Coping (T2)		.363	.165	.040	.686	.268	.439	<.001
Introjected Regulation	GD risk T1 → GD risk T2	1.807	.899	.679	.475	3.138	1.316	2.552	<.001
	IntrReg T1 → IntrReg T2	.621	.513	.172	.284	.959	.561	.694	<.001
	IntrReg T1 → GD risk T2	-.114	-.054	.291	-.684	.455	-.396	.094	.301
	**GD risk T1 → IntrReg T2**	**.084**	**.080**	**.283**	**.041**	**.443**	**-.023**	**.172**	**.030**
	Correlation GD ↔ IntrReg (T1)		.691	.072	.550	.831	.652	.732	<.001
	Residual correlation GD ↔ IntrReg (T2)		.738	.144	.456	1.020	.665	.933	<.001
Escape	GD risk T1 → GD risk T2	1.887	.928	.676	.555	3.205	1.293	2.74	<.001
	Escape T1 → Escape T2	1.438	.830	.289	.872	2.004	1.287	1.635	<.001
	Escape T1 → GD risk T2	-.236	-.114	.283	-.790	.320	-.712	.012	.085
	**GD risk T1 → Escape T2**	**-.043**	**-.025**	**.199**	**-.434**	**.347**	**-.208**	**.044**	**.442**
	Correlation GD ↔ Escape (T1)		.596	.089	.422	.769	.524	.695	<.001
	Residual correlation GD ↔ Escape (T2)		.792	.200	.402	1.176	.672	1.059	<.001

*B* – mean unstandardized regression coefficient across allocations; *Beta* – mean standardized regression coefficient across allocations; *SE* – standard error of pooled unstandardized estimates; *LLCI* and *ULCI* – lower and upper level of 95% confidence interval of pooled unstandardized estimates; *Min* and *Max* - minimum and maximum unstandardized estimate across parcel allocations.

Boldface indicates parameters directly testing the primary study hypotheses. All other rows report additional model outputs included for completeness. GD, Gaming Disorder; T1, Time 1; T2, Time 2.

## 4 Discussion

The aim of the study was to examine prospective associations between motivations for gaming and the risk of GD among adolescents. In line with H1, all motivational factors showed positive associations with GD at both time points. Based on the cross-lagged panel analysis, the hypothesis related to gratification-based motivations (H2) was supported, whereas the hypothesis related to compensation-related motivations (H3) was partially supported. Both Fantasy and Identity measured at T1 were prospectively associated with the risk of GD at T2, suggesting that gratification motivations are a risk factor for the development of problematic gaming. GD risk measured at T1 was prospectively associated with higher levels of two of the compensation-related motivations at T2, namely Coping and Introjected Regulation, but not Escape.

### 4.1 Correlational associations (H1)

Consistent with Hypothesis 1, all motivational factors—including Coping, Escape, Introjected Regulation, Fantasy, and Identity—were positively correlated with GD risk at both measurement points. These findings confirm previous cross-sectional studies demonstrating robust associations between immersion/escapism motives and problematic gaming (2, 8, 30). The strongest correlations were observed for Introjected Regulation and Fantasy, highlighting that both compensatory and gratification-related motives are tightly linked to GD risk in adolescence. This pattern underscores the concurrent interplay between motivations and problematic gaming, although correlations alone cannot determine their temporal direction.

### 4.2 Gratification motives as predictors of GD (H2)

Both Fantasy and Identity at Time 1 were prospectively associated with higher GD risk at Time 2, after accounting for GD risk at Time 1, thus supporting Hypothesis 2. These findings indicate temporal ordering within the present sample but do not demonstrate that gratification-related motives initiate or cause increases in GD symptoms. They are consistent with the possibility that gratification-related motives precede higher subsequent GD symptom severity, although the processes underlying these associations were not tested. Moreover, because a two-wave cross-lagged model does not separate stable between-person differences from within-person processes, the observed paths cannot be interpreted as evidence of individual-level change.

GD risk at Time 1 was prospectively associated with lower Identity motivation at Time 2 after accounting for its baseline level. Since adolescence is a critical period for identity exploration (31, 32) and analogous study on emerging adults did not show similar effect (13), this specific result may be a signal for further studies on dynamics of identity motivation and gaming among adolescents. At the moment, this finding should be interpreted specifically in relation to the GMI Identity subscale, which assesses the extent to which gaming is experienced as personally meaningful or identity-related. It does not measure general identity coherence or adolescent identity development. One possible interpretation is that higher GD risk is followed by weaker endorsement of identity-related reasons for gaming, potentially reflecting a change in how adolescents relate to gaming as symptoms become more severe. However, the psychological processes underlying this association were not directly assessed and should be examined using dedicated measures of identity and self-concept. In contrast, the reverse path from GD risk at Time 1 to Fantasy at Time 2 did not reach statistical significance (*b* = .162, *p* = .063).

### 4.3 Prospective associations of GD risk with compensation motives (H3)

Partially supporting Hypothesis 3, the risk of GD at Time 1 was prospectively associated with higher Time 2 levels of Coping and Introjected Regulation after accounting for their respective baseline levels. However, no prospective association was observed for Escape motivation. These findings suggest a nuanced relationship between disorder risk and compensatory motives in adolescents. From the perspective of Goal Systems Theory (15), gaming can be understood as a means linked to multiple goals. The observed pattern is consistent with the possibility that, among adolescents with higher GD risk, gaming may be more strongly linked to coping with distress or responding to internalized pressures. This interpretation is also compatible with models proposing that compensatory motives may become more salient alongside greater GD risk (3, 19, 20). However, the present findings do not demonstrate changes in goal–means relationships, or that these motives emerge as consequences of GD risk.

The null finding for Escape may suggest not that this motive is irrelevant, but that it operates differently from other compensation-related motivations (33, 34). Unlike Coping and Introjected Regulation, Escape may be more sensitive to short-term fluctuations in distress, which could make its prospective association with GD symptoms less readily detectable over a three-month interval (35, 36). This interpretation remains tentative and requires examination using more frequent measurement occasions.

## 5 Theoretical implications

The findings provide partial support for the I-PACE model (9, 10), showing motive-specific prospective associations between gaming motivations and GD risk. Gratification motives (Fantasy, Identity) at Time 1 were associated with GD risk at Time 2, while GD risk at Time 1 was associated with higher Time 2 levels of Coping and Introjected Regulation, but not Escape. This pattern is consistent with different temporal relationships between gratification-oriented and compensation-oriented motives and GD risk, but it should be replicated.

The negative path from GD risk to Identity suggests that prospective relationships may also differ between individual gratification-oriented motives. The selective associations with Coping and Introjected Regulation, but not Escape, similarly indicate heterogeneity among compensation-oriented motives. Future research should test whether these patterns reflect within-person processes and how they relate to identity formation, emotion regulation capacity, and neurobiological maturation, into models of behavioral addiction.

## 6 Practical implications

The findings provide preliminary directions for future prevention and intervention research. Their practical relevance should be evaluated in larger and clinically characterized samples, with longer follow-up periods and designs capable of distinguishing within-person processes from stable between-person differences.

First, future studies could examine whether Fantasy and Identity motivations provide incremental information about subsequent GD symptoms beyond established risk indicators. Such research could determine whether these motives contribute to reliable, multi-indicator approaches to identifying adolescents who may require additional support.

Second, the finding that GD risk was prospectively associated with subsequent Coping and Introjected Regulation identifies these motives as potential processes for future intervention research. Controlled studies could test whether interventions focused on emotion regulation, alternative coping strategies, or internalized pressures produce changes in these motives and in GD symptoms. If these associations are replicated and these motives are shown to represent modifiable mechanisms, cognitive-behavioral therapy could provide a suitable framework for addressing them by strengthening alternative coping strategies, developing emotion-regulation skills, and examining internalized pressures related to gaming.

Third, the absence of effects for Escape motivation suggests that each compensatory motivation should be treated individually rather than assuming uniform patterns. Future research comparing these motives may help determine their respective relevance to prevention and intervention. If this differentiation is replicated and shown to be clinically meaningful, it could support motive-specific assessment and the evaluation of interventions tailored to the distinct roles of individual gaming motives.

## 7 Limitations

The present study has several limitations that should be considered when interpreting the findings. First, a substantial portion of participants could not be matched across waves because of either attrition or unsuccessful matching based on self-generated anonymous codes, which reduced the effective longitudinal sample size and may have introduced bias related to participant retention. Although participants retained and not retained in the longitudinal sample did not differ on the measured baseline variables, selection related to unmeasured characteristics cannot be excluded. Second, all variables were assessed through self-report questionnaires, which might be affected by response bias, memory inaccuracies, or social desirability effects, despite the inclusion of honesty and attention checks. Moreover, GD risk was assessed using a screening instrument rather than a clinical diagnostic assessment Third, the sample consisted exclusively of high school students, and therefore the findings cannot be generalized to other populations of adolescents. Fourth, data were collected from students attending a single high school within a single national context, which may limit the generalizability of the observed relationships.

In addition, because specific classroom membership was not recorded, potential non-independence of observations due to classroom-level clustering could not be assessed.

Fifth, the study included only two measurement occasions. Consequently, the traditional cross-lagged models could not distinguish stable between-person differences from within-person processes, and accounting for baseline levels should not be interpreted as demonstrating individual-level change (37). Although the models establish temporal ordering within the three-month interval, temporal precedence alone does not establish causality, as the observed paths may reflect stable individual differences, unmeasured confounding, or other processes not represented in the models. The findings should therefore be interpreted as prospective associations rather than demonstrated developmental mechanisms.

Sixth, the three-month interval should also be considered when interpreting the findings. Although it was theoretically consistent with a dynamic view of motivation and followed the design of previous study (13), it may have been too short to capture slower changes in gaming motivations or GD risk and does not permit conclusions about longer-term developmental trajectories. Longer intervals are not inherently more appropriate in cross-lagged research because the magnitude of cross-lagged associations depends on the temporal characteristics and stability of the constructs under investigation (38). Nevertheless, because the present study included only a single three-month lag, it cannot establish whether this interval was optimal. Future studies should incorporate additional waves and multiple time intervals to compare prospective associations across shorter and longer temporal windows.

Finally, despite additional variables were collected as part of the broader research project, they were not included in the present models. Given the sample size and model complexity, fully adjusted analyses were not estimated; consequently, the robustness of the findings to these potential covariates remains unknown and should be examined in larger samples.

## 8 Future research

Future studies should include additional measurement waves to better capture the temporal dynamics of the relationships between gaming motivations and GD, and to test possible associations operating in both directions. Larger and better-tracked longitudinal samples are needed to reduce attrition bias and strengthen the reliability of cross-lagged findings. To overcome the limitations of self-report measures, future research could integrate quantitative and qualitative methods, such as behavioral indicators (e.g., playtime logs) or interviews exploring subjective gaming experiences. Extending recruitment beyond high school populations would allow examination of developmental and contextual variations in gaming motivations. Finally, cross-cultural longitudinal projects are warranted to determine whether the gratification- and compensation-oriented patterns identified here generalize across different sociocultural settings.

## 9 Conclusions

This study found that gratification-related motives, particularly Fantasy and Identity, at Time 1 were prospectively associated with higher GD risk at Time 2 among high school students, whereas GD risk at Time 1 was prospectively associated with higher Coping and Introjected Regulation and lower Identity at Time 2, but not with subsequent Fantasy or Escape. These results identify motive-specific temporal associations but do not establish that gratification-based motivations drive disorder onset or that GD risk causes subsequent changes in gaming motives. Because the two-wave cross-lagged model cannot distinguish stable between-person differences from within-person processes, the findings should not be interpreted as demonstrating developmental mechanisms. The pattern of prospective associations differed across motives and directions, indicating that the relationships between specific gaming motives and GD risk were not uniform. Further longitudinal and cross-cultural research using larger samples, additional measurement occasions, and models that separate between-person and within-person variance is needed to assess the robustness of these associations and determine whether they may eventually inform prevention research.

## Data Availability

The datasets presented in this study can be found in online repositories. The names of the repository/repositories and accession number(s) can be found below: https://osf.io/d6ujs/files/osfstorage/68ffb82b6ded2b8f49c407fe.
