# Supplementary material for: Gratification vs. compensation: longitudinal evidence for the shifting motivations behind adolescent gaming disorder
Source: Front Psychiatry. 2026 Oct 23;17:1938938. doi: 10.3389/fpsyt.2026.1938938 (PMC13739800; doi:10.3389/fpsyt.2026.1938938)
Supplement: Supplementary file 1 [file Table1.docx]

**Figure S1**

*Participant flow and cross-wave matching across the two study waves*

**Table S2**

*Descriptive Statistics*

|  | *M* | *Me* | *SD* | *Sk.* | *Kurt.* | *Min* | *Max* |
| --- | --- | --- | --- | --- | --- | --- | --- |
| GD T1 | 1.82 | 1.78 | .55 | 1.10 | 1.93 | 1.00 | 3.89 |
| GD T2 | 1.79 | 1.72 | .57 | .44 | -.84 | 1.00 | 3.00 |
| Fantasy T1 | 3.02 | 2.50 | 1.64 | .44 | -1.04 | 1.00 | 6.50 |
| Fantasy T2 | 3.12 | 3.00 | 1.67 | .44 | -.92 | 1.00 | 6.75 |
| Identity T1 | 2.59 | 2.40 | 1.40 | .73 | -.45 | 1.00 | 6.40 |
| Identity T2 | 2.60 | 2.20 | 1.42 | .74 | -.47 | 1.00 | 6.40 |
| Coping T1 | 3.97 | 4.00 | 1.50 | .12 | -.63 | 1.00 | 7.00 |
| Coping T2 | 3.92 | 4.00 | 1.63 | -.07 | -.98 | 1.00 | 7.00 |
| Introjected Regulation T1 | 1.64 | 1.33 | .82 | 1.36 | 1.41 | 1.00 | 4.67 |
| Introjected Regulation T2 | 1.81 | 1.33 | 1.04 | 1.21 | .61 | 1.00 | 5.33 |
| Escape T1 | 3.25 | 3.00 | 1.71 | .66 | -.53 | 1.00 | 7.00 |
| Escape T2 | 3.31 | 3.25 | 1.77 | .45 | -.84 | 1.00 | 7.00 |
